# In silico identification of thiostrepton as an inhibitor of cancer stem cell growth and an enhancer for chemotherapy in non–small‐cell lung cancer

**DOI:** 10.1111/jcmm.14689

**Published:** 2019-10-22

**Authors:** Tse‐Hung Huang, Alexander T. H. Wu, Tai‐Shan Cheng, Kuan‐Ting Lin, Chia‐Jou Lai, Hao‐Wen Hsieh, Peter Mu‐Hsin Chang, Cheng‐Wen Wu, Chi‐Ying F. Huang, Kuan‐Yu Chen

**Affiliations:** ^1^ Department of Traditional Chinese Medicine Chang Gung Memorial Hospital Keelung Taiwan; ^2^ School of Traditional Chinese Medicine Chang Gung University Taoyuan Taiwan; ^3^ School of Nursing National Taipei University of Nursing and Health Sciences Taipei Taiwan; ^4^ Graduate Institute of Health Industry Technology Chang Gung University of Science and Technology Taoyuan Taiwan; ^5^ Research Center for Chinese Herbal Medicine Chang Gung University of Science and Technology Taoyuan Taiwan; ^6^ The Ph.D. Program for Translational Medicine College of Medical Science and Technology Taipei Medical University Taipei Taiwan; ^7^ Institute of Biopharmaceutical Sciences National Yang‐Ming University Taipei Taiwan; ^8^ Cold Spring Harbor Laboratory Cold Spring Harbor NY USA; ^9^ Institute of Clinical Medicine National Yang‐Ming University Taipei Taiwan; ^10^ Department of Oncology Taipei Veterans General Hospital Taipei Taiwan; ^11^ Faculty of Medicine National Yang‐Ming University Taipei Taiwan; ^12^ Institute of Biomedical Sciences Academia Sinica Taipei Taiwan; ^13^ Department of Biochemistry College of Medicine Kaohsiung Medical University Kaohsiung Taiwan; ^14^ Division of Pulmonary Medicine Department of Internal Medicine National Taiwan University Hospital and College of Medicine National Taiwan University Taipei Taiwan

**Keywords:** cancer stem cell, epithelial‐to‐mesenchymal transition, miR‐98, non–small‐cell lung cancer, thiostrepton

## Abstract

Cancer stem cells (CSCs) play an important role in cancer treatment resistance and disease progression. Identifying an effective anti‐CSC agent may lead to improved disease control. We used CSC‐associated gene signatures to identify drug candidates that may inhibit CSC growth by reversing the CSC gene signature. Thiostrepton, a natural cyclic oligopeptide antibiotic, was the top‐ranked candidate. In non–small‐cell lung cancer (NSCLC) cells, thiostrepton inhibited CSC growth in vitro and reduced protein expression of cancer stemness markers, including CD133, Nanog and Oct4A. In addition, metastasis‐associated Src tyrosine kinase signalling, cell migration and epithelial‐to‐mesenchymal transition (EMT) were all inhibited by thiostrepton. Mechanistically, thiostrepton treatment led to elevated levels of tumour suppressor miR‐98. Thiostrepton combined with gemcitabine synergistically suppressed NSCLC cell growth and induced apoptosis. The inhibition of NSCLC tumours and CSC growth by thiostrepton was also demonstrated in vivo. Our findings indicate that thiostrepton, an established drug identified in silico, is an inhibitor of CSC growth and a potential enhancer of chemotherapy in NSCLC.

## 1 INTRODUCTION

Lung cancer is the leading cause of cancer‐related death worldwide.^1^ The five‐year survival rate is approximately 18% for patients with lung cancer and 3.9% for those with advanced stages^2^ of the disease, lower than the survival rates for colon (65%), female breast (90%) and prostate (99%) cancers.^3^ Non–small‐cell lung cancer (NSCLC) accounts for approximately 85% of all lung cancers. Chemotherapies, including pemetrexed or gemcitabine in combination with platinum, are frequently used as the first‐line therapy for advanced NSCLC.^4^, ^5^, ^6^ However, current chemotherapy regimens do not provide long‐term disease control in most patients with NSCLC.^7^, ^8^ Those who receive chemotherapy eventually encounter drug resistance and disease progression.^9^, ^10^ Alternative therapeutic strategies are urgently needed.

Cancer stem cells (CSCs) play an important role in cancer recurrence, progression and drug resistance.^11^, ^12^, ^13^ Lung CSCs were first identified as CD133^+^ cells^14^ and isolated in established NSCLC cell lines,^15^, ^16^ forming floating spheres in serum‐free conditions. These CD133^+^ lung cancer spheres exhibited self‐renewal abilities, stress/drug resistance, epithelial‐to‐mesenchymal (EMT) potential and the ability to recapitulate tumour heterogeneity in vivo.^14^, ^15^, ^16^, ^17^, ^18^ The drug resistance may be due to features related to the stem cell pathway, expression of high‐level ATP‐binding cassette transporters and specific surface biomarkers.^19^ Identifying agents to eliminate CSCs has become an important issue for anti‐cancer drug development.

Developing an entirely new drug is expensive and time‐consuming. Drug repurposing offers an alternative strategy for anti‐cancer drug development, which requires analysing biological and medical information for a huge number of drugs.^20^ The Connectivity Map (CMap, http://www.broad.mit.edu/cmap/),^21^ which stores expression profiles of diseases, genes and chemicals, provides a tool for making inferences based on a query and the internal profiles. We hypothesized that drugs with the ability to reverse the expression of CSC‐like gene signatures may inhibit CSC growth and could prove beneficial in the treatment of lung cancer. We tested the top‐ranked agent identified by CMap for reversing potential CSC gene signatures for anti‐CSC abilities in vitro and in vivo. The synergistic effect of identified agents and existing chemotherapies on tumour growth inhibition was also examined.

Thiostrepton, a macrocyclic thiopeptide antibiotic, belongs to the thiopeptide family of highly modified macrocyclic peptides, which are produced as secondary metabolites by actinomycetes of the genus *Streptomyces*.^22^ Thiostrepton interacts directly with forkhead box M1 (FOXM1) and inhibits binding with genomic target sites.^23^ Thiostrepton also inhibits growth and induces apoptosis in human cancer cells by inhibiting FOXM1 expression.^24^ Based on CMap analysis, we identified thiostrepton as a candidate to be an anti‐CSC agent. We also provided evidence that thiostrepton can suppress cancer cell proliferation, migration and CSC‐like properties in vitro, as well as inhibit tumorigenesis in vivo. More importantly, thiostrepton combined with gemcitabine inhibited NSCLC cell growth synergistically. Collectively, our findings suggest thiostrepton can serve as an anti‐CSC drug and play a beneficial role in lung cancer treatment.

## 2 MATERIALS AND METHODS

### 2.1 Cell lines and chemicals

A549, NCI‐H441 and CL141 are lung adenocarcinoma cell lines with wild‐type *EGFR*. CL152 is a lung squamous carcinoma cell line. H1299 is an NSCLC cell line, and H460 is large cell lung carcinoma cell line. A549‐ON cells are A549 cells co‐overexpressed Oct‐4A and Nanog.^25^ H460 cells were infected by a CD133 P1 promoter‐driven GFP reporter lentivirus and cultured in RPMI‐1640 supplemented with 10% foetal bovine serum (FBS) (GIBCO).^26^ CD133^+^GFP^+^H460 cells were sorted by using a FACSAria cell sorter (BD Biosciences). The other cell lines were maintained in RPMI medium and supplemented with 10% FBS (GIBCO), 2 mmol/L l‐glutamine, 100 U/mL penicillin and 100 μg/mL streptomycin (GIBCO). For cell culture experiments, a 10 mmol/L thiostrepton stock solution was dissolved in dimethyl sulfoxide (DMSO; Sigma). Pemetrexed, gefitinib and gemcitabine were purchased from LC Laboratories, while thiostrepton and cisplatin were acquired from Sigma and Selleckchem, respectively.

### 2.2 L1000 expression profiling

Gene expression profiles were obtained from cancer cells treated with perturbagens, including small molecules and Chinese herbal medicines, in triplicate for 6 hours, followed by L1000 expression profiling^21^, ^27^ by Genometry Inc In brief, cells were lysed after 6 hours of treatment in triplicate, and mRNA transcripts from whole cell lysates were rapidly captured by oligo‐dT. The cDNAs generated by reverse transcription from mRNA were amplified through ligation‐mediated polymerase chain reaction (PCR). The PCR amplicon was then hybridized to bar‐coded Luminex beads to display the expression values of specific genes. The cDNA was annealed with specific probes for 978 landmark genes. A list of overexpressed and underexpressed probe sets was obtained using a *t* test rank order.

### 2.3 Connectivity scoring by gene set enrichment analysis using CMap

Intensity values of gene expression profiles were first converted to robust z‐scores using the l1ktools downloaded from the CMap/Library of Integrated Network‐based Cellular Signatures (LINCS) project of the NIH Common Fund programme. Integrated Network‐based Cellular Signatures has been replaced by the CLUE platform as of February, 2017. The new analytical tool can be accessed from https://clue.io. A Perl script was used to calculate the connectivity score for each gene expression profile against the anti‐CSC or CSC gene signature.^21^ The anti‐CSC gene signature was identified using GEO2R for differentially expressed genes in the study of, for example, the Gene Expression Omnibus^28^ gene signature GSE18150.^29^

The 11 641 profiles in our collection were ranked by connectivity scores and used as the input of the ranking matrix for gene set enrichment analysis (GSEA). Thiostrepton profiles (including repeats and treatments in different cancer cells) were grouped together and used as the input gene set for GSEA to map the ranking matrix and calculate the enrichment scores. Normalized enrichment scores were obtained from 1000 permutations of gene sets. Some of the gene signatures (eg GSE18931 30) were obtained as CSC gene signatures. We scored the negative enrichment. Finally, for CMap/LINCS analysis, the query‐gene signature was uploaded to LINCS Web Apps to obtain score_best4 scores of the perturbagens in the database.

### 2.4 Colony formation assay

Non–small‐cell lung cancer cells were seeded in 6‐well plates at a density of 600 cells per well and cultured for 14 days. Thiostrepton was added 24 hours after seeding. The culture medium with thiostrepton was renewed every 4 days. Following the treatments, cells were washed with phosphate‐buffered saline (PBS), and the colonies were fixed in a methanol‐acetic acid fixing solution with a ratio of 3:1 and stained with 0.5% crystal violet solution in methanol. After carefully removing the crystal violet solution and rinsing with tap water, the colonies were counted manually. Each experiment was performed independently, in triplicate, at least twice.

### 2.5 Cytotoxicity assay and drug combination analysis

Cells were seeded in 96‐well plates at a density of 2000 cells per well in triplicate. The cells were treated with indicated agents for 48 hours on the second day to ensure adequate plating efficiency and cell vitality. Cells were treated with different concentrations of thiostrepton, pemetrexed, cisplatin, gemcitabine and gefitinib or a non‐fixed‐ratio combination of thiostrepton and one of the anti‐cancer agents.

The cytotoxicity was assessed by using a sulforhodamine B (SRB) assay.^31^ Briefly, the medium was discarded, and the adherent cells were fixed with 100 μL of cold 10% trichloroacetic acid (w/v) in each well for 1 hour at 4°C. Cells were stained after fixing with 100 μL/well of 0.4% (w/v, in 1% acetic acid) SRB solution for 30 minutes at room temperature and then washed five times with 1% acetic acid. After air‐drying, 100 μL of 10 mmol/L Tris base was added to each well and the absorbance was read at 546 nm. Cytotoxicity was defined as the percentage of cells in the drug‐treated wells relative to the cell numbers in the solvent‐only control (set to 100%). Each experiment was performed independently, in triplicate, at least twice, and the cytotoxicity was presented as the mean ± standard deviation.

The synergy associated with inducing cytotoxicity among different drug combinations was evaluated by analysis of the median‐dose effect and calculation of the combination index (CI) using commercially available software of Chou and Talalay software (CompuSyn).^32^, ^33^ According to the recommendations of this methodology, CI values of less or greater than 1 indicated synergism or antagonism, respectively. A value of 1 indicated an additive effect.

### 2.6 Cell migration assay

Cell culture inserts (Millipore) were placed in 24‐well plates. Serum‐starved cells (2 × 10^5^ cells) were seeded in the upper chambers of the transwell with 200 μL of serum‐free medium, in the presence of the vehicle (DMSO) or thiostrepton (5 μmol/L). The lower chambers were filled with 750 μL of medium containing 10% FBS as a chemo‐attractant. After incubation for 16 hours, cells were fixed in 4% formaldehyde and stained with GIEMSA (Sigma). After washing, the non‐penetrating cells on the inner surfaces of the upper chambers were wiped off with cotton swabs. The penetrated cells were photographed and counted using a light microscope. Each assay was performed in triplicate.

### 2.7 Immunoblotting

After treatments, cells were lysed in a lysis buffer. Total protein contents were isolated and subjected to SDS polyacrylamide gel electrophoresis and electro‐transferred onto polyvinylidene fluoride membranes (Millipore). Immunoblotting was performed using primary antibodies, including β‐catenin (Cell Signaling Technology), c‐Myc (Cell Signaling Technology), CD44 (Cell Signaling Technology), CD133 (GeneTex), Oct‐4A (GeneTex), Sox2 (Cell Signaling Technology), FOXM1 (Abcam), E‐cadherin (Cell Signaling Technology), Src (Cell Signaling Technology), phosphorylated Src (Tyr416, Cell Signaling Technology), caspase‐8 (Cell Signaling Technology), caspase‐9 (Cell Signaling Technology), caspase‐3 (Cell Signaling Technology) and cleaved PARP (Cell Signaling Technology). GAPDH (Cell Signaling Technology), α‐tubulin (Cell Signaling Technology) or β‐actin (GeneTex) acted as an internal control. Protein detection was performed by using an enhanced chemiluminescence (ECL™) method and the Luminescence Imaging System (LAS‐4000™, Fuji Photo Film Co., Ltd).

### 2.8 Assays for cancer stemness characteristics

The assay of ALDH1 activity is frequently used to define lung cancer stem cell populations.^34^ Aldefluor assays were performed according to the manufacturer's guidelines (StemCell Technologies). Briefly, a single cell suspension obtained from cell cultures was incubated in Aldefluor assay buffer containing an ALDH substrate (bodipy‐aminoacetaldehyde, BAAA) for 50 minutes at 37°C. As a negative control, a fraction of cells from each sample was incubated under identical conditions in the presence of an ALDH inhibitor (diethylaminobenzaldehyde, DEAB). Flow cytometry was used to detect the ALDH‐positive cell population.

A tumour sphere formation assay was carried out to evaluate cancer stemness. Lung cancer cells were seeded in 6‐well ultralow‐attachment plates (Corning Inc) at a density of 2000 cells/mL in a serum‐deprived culture medium consisting of DMEM/F12 supplemented with 1% N2 Supplement (GIBCO), 20 ng/mL basic fibroblast growth factor (Sigma), 20 ng/mL epidermal growth factor (GIBCO), 100 U/mL penicillin and 100 μg/mL streptomycin (GIBCO) at 37°C in a humidified atmosphere of 95% air and 5% CO_2_. Tumour spheres were counted after harvest using a Countess™ (GIBCO) automated cell counter.

### 2.9 Quantitative PCR analyses and microRNA assays

All RNA‐related experiments were performed by using kits purchased from QIAGEN (Taiwan) and following the instructions provided by the vendor. Total RNA was isolated, quantified and reverse transcribed into cDNA. The primers of different microRNAs tested in this study for microRNA quantitative real‐time PCR assays were also purchased from QIAGEN. Up‐regulation and down‐regulation of miRNAs were performed by transfecting cells with miRNA precursors or anti‐miRNAs, respectively. Fifty nanomoles of miScript mimic and inhibitors were transfected into lung cancer cells by using the lipofectamine 2000 reagent (Thermo Fisher Scientific). Total RNA and protein were isolated 48 hours after treatments to determine the effects on target‐protein expression profiles.

### 2.10 Examination of anti‐lung cancer effects mediated by thiostrepton in vivo

The inhibitory effect of thiostrepton on tumour growth was evaluated by using a mouse subcutaneous tumour xenograft model. Human lung adenocarcinoma cells (NCI‐H441, purchased from ATCC) were injected subcutaneously in the right flank of non‐obese diabetic/severe combined immunodeficient (NOD/SCID) mice (female, 4‐6 weeks old) at 10^6^ cells/injection. Once the tumour became palpable, the tumour‐bearing mice were randomly assigned to a thiostrepton group (5 mg/kg, 5 days/wk, intraperitoneal injection) or a control group (DMSO vehicle). Over a period of 4 weeks, the tumour sizes in both groups were measured weekly with standard calipers. The in vivo tracking of tumour growth was then determined and presented as the fold change in tumour volume over time. All animal experiments were approved by the Taipei Medical University Animal Center (Protocol number: LAC‐2013‐0086). The animals were humanely killed by cervical dislocation to minimize the suffering. All tumour samples were harvested for further analyses.

## 3 RESULTS

### 3.1 Identification of thiostrepton as a potential anti‐CSC agent using the Connectivity Map database

**Figure 1:**
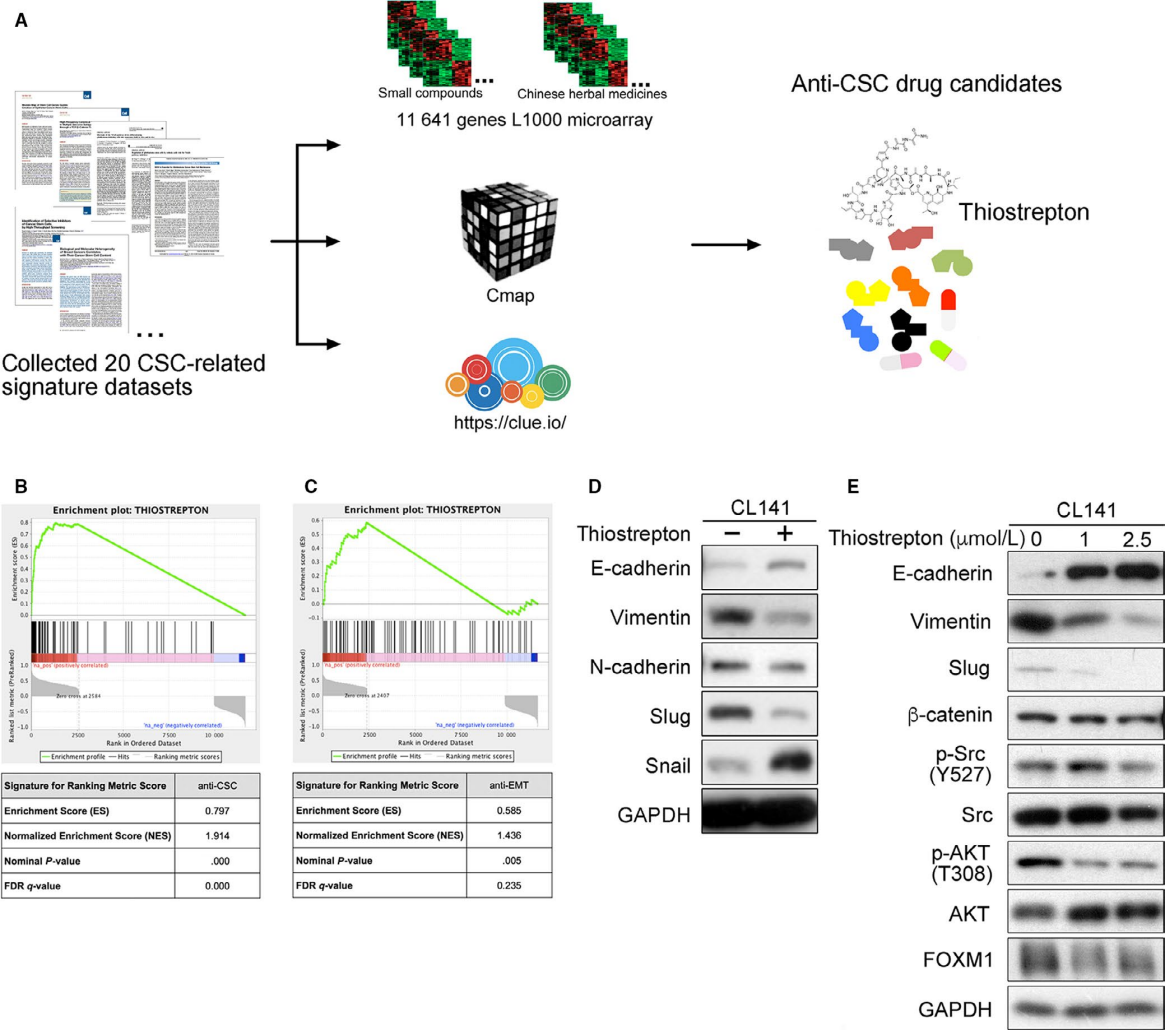
Identification of thiostrepton as an anti‐cancer stem cell agent using genomic approaches. A, Flowchart of array analysis and prediction of potential anti‐CSC agents in NSCLC. B, Pre‐ranked GSEA of 81 gene expression profiles of thiostrepton among the 11 641 L1000 gene expression profiles. The ranking metric scores were connectivity scores against the anti‐CSC signature of DZNep (GSE18150). The hits (black vertical lines) are thiostrepton profiles. C, The anti‐EMT signature (GSE17215) of salinomycin was used to generate connectivity scores for ranking metric scores. D, A Western blotting assay indicated that thiostrepton treatment (2.5 μmol/L) enhanced E‐cadherin expression and reduced Slug expression in CL141 cells. E, Thiostrepton treatment reduced vimentin and Slug expression, inhibited Src and AKT activation and reduced FOXM1 expression in a dose‐dependent manner in CL141 cells

We compared 20 different published data sets (Table S1) and employed CSC‐related gene expression profiles as inputs to query 11 641 L1000‐based gene expression profiles. Thiostrepton was identified as a candidate that could significantly reverse lung cancer gene signatures (Figure 1A). As an example, GSE18150 was originally generated from cells treated with DZNep by disrupting *EZH2* and impairing CSC self‐renewal.^29^ Thiostrepton profiles, including treatments in different cancer cells in triplicate, were grouped together and used as the input gene set for GSEA to map the ranking matrix and calculate an enrichment score. The anti‐CSC gene signature from GSE18150 and cancer cells treated with thiostrepton had a strong enrichment score in our 11 641 L1000 assays (normalized enrichment score = 1.914, *P*‐value <.0001, Figure 1B). The majority of thiostrepton profiles had a positive connectivity score, and only a few had a score of zero. This indicated a strong positive connectivity between thiostrepton and the anti‐CSC gene signature, meaning thiostrepton is a potential anti‐CSC agent.

When the anti‐CSC gene signature GSE18150 determined by GEO2R was used to query the CMap 2.0 (a database containing 6100 gene expression profiles), thiostrepton was ranked as the top candidate (enrichment score = 0.797, *P*‐value <.001; Figure 1B). In results from the query of the LINCS database, which contained more than 1.3 million gene expression profiles, thiostrepton was ranked as the 22nd among drugs with positive similarity scores (score_best4 = 97.368).

The human normal mammary stem cell (hNMSC) gene expression signature GSE18931, a CSC gene signature, can stratify biological and molecular features in tumours (Table S2). The degree of cells expressing hNMSC markers may reflect the CSC content of a tumour.^30^ L1000 profiles with a negative connectivity score (normalized enrichment score = −2.0146; *P*‐value <.0001; false discover rate <0.0001) indicated that thiostrepton treatment may reverse gene signatures of GSE18931. Similarly, when GSE18931 was used to query the LINCS database, the results showed that thiostrepton had a score_best4 of −93.176 among the compounds with negative similarity scores. This strong concordance across different perturbagen databases indicated that thiostrepton is likely to have anti‐CSC abilities.

In addition, our GSEA showed that thiostrepton has significant connectivity with the anti‐EMT gene signature of salinomycin (GSE17215) with specific toxicity for epithelial CSCs^35^ (normalized enrichment score = 1.436; *P*‐value = .005, Figure 1C). Further validation was demonstrated by the up‐regulated expression of E‐cadherin and down‐regulated expression of Slug and vimentin in thiostrepton‐treated CL141 cells (Figure 1D). Moreover, treatment with thiostrepton not only distinctly reduced FOXM1 expression but also reduced vimentin and Slug expression and inhibited Src and AKT activation in CL141 cells in a dose‐dependent manner (Figure 1E).

### 3.2 Thiostrepton suppresses NSCLC cell growth, clonogenicity and migration

**Figure 2:**
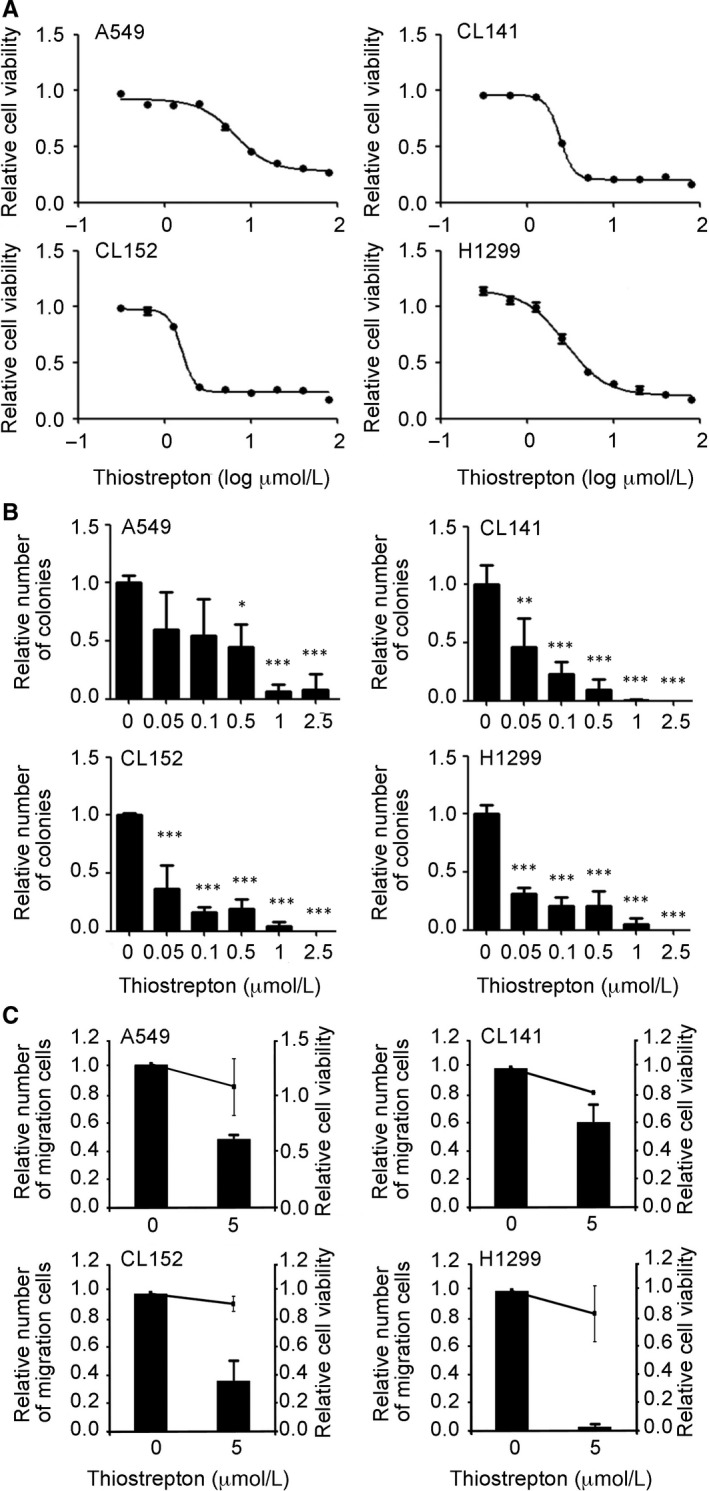
Thiostrepton treatment suppresses NSCLC tumorigenesis. Thiostrepton treatment significantly suppressed NSCLC cell viability (A) and colony formation (B) in A549, H1299, CL141 and CL152 cells. C, Thiostrepton inhibited NSCLC cell migration. The bars indicate relative migratory ability (left *y*‐axis), which is compared with the vehicle (control; 0). Relative cell viability (right *y*‐axis) is indicated by solid lines. These data suggest that the decreased cell migration by thiostrepton was not associated with the reduction of cell viability. **P < .01, ***P < .001

Non–small‐cell lung cancer cells were treated with thiostrepton. Figure 2A,B shows that thiostrepton suppressed cellular viability and colony‐forming ability in a dose‐dependent manner. The half‐maximal inhibitory concentration (IC_50_) of thiostrepton in the clonogenic assay was approximately 0.05 μmol/L, which was distinctly lower than that of cytotoxic effects (Table S3). In addition, treatment with thiostrepton significantly reduced the migration capability of A549, CL141, CL152 and H1299 cells (Figure 2C).

A previous study demonstrated enhanced sphere‐forming ability, cisplatin resistance and migration ability in CD133^+^ H460 cells.^26^ To verify the potential anti‐CSC effect of thiostrepton, we used H460 cells expressing GFP driven by a CD133 promoter for cytotoxicity analysis. Both parental H460 and CD133^+^ H460 cells were then subjected to an SRB assay. We found that the CD133^+^ H460 cells (IC_50_ = 1.7 μmol/L) were more sensitive to thiostrepton than the parental H460 cells (IC_50_ = 6.9 μmol/L; Table S3).

### 3.3 Thiostrepton suppresses CSC properties in NSCLC cells

**Figure 3:**
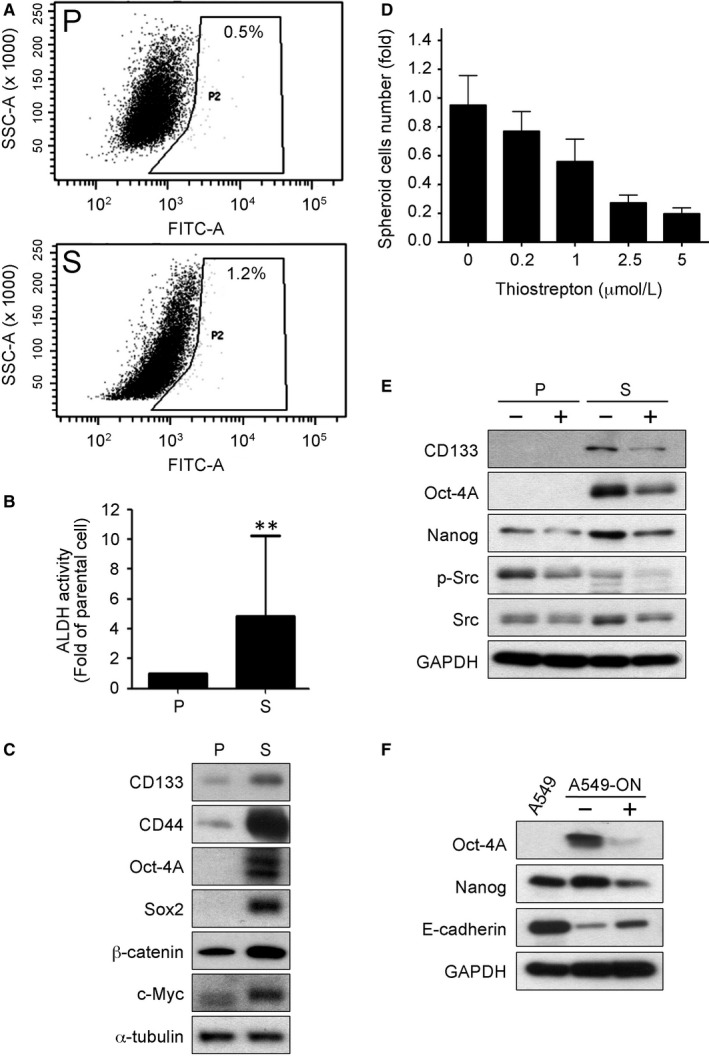
Thiostrepton inhibits cancer stem cell properties. A, Flow cytometric analysis demonstrated that CL141 spheres (S) exhibited a higher percentage of ALDH1^+^ cells compared with the CL141 parental cells (P). B, Quantitative presentation of the relative ALDH activity in CL141 spheres (S) compared with CL141 parental cells (P). C, Western blotting assay indicated that CL141 spheres (S) expressed a significantly higher level of CD133, CD44, Oct‐4A and Sox2 (CSC markers) as well as β‐catenin and c‐Myc (oncogenic markers) compared with their parental counterparts (P). D, Thiostrepton suppressed tumour sphere formation in CL141 cells in a dose‐dependent manner. E, Comparative western blotting assay of parental cells (P) and spheres (S) showed that thiostrepton treatment led to decreased expression of CD133, Oct‐4A, Nanog (CSC markers) and metastasis‐associated Src signalling. F, In the Oct‐4A/Nanog overexpressing A549 (A549‐ON) cells, thiostrepton treatment significantly decreased the expression of both cancer stem cell markers, Oct‐4A and Nanog, and increased E‐cadherin expression

For in vitro validation, tumour spheres of CL141 cells were generated under serum‐free conditions and CSC properties were examined in these tumour spheres. By using an Aldefluor assay, we found CL141 tumour spheres were enriched with ALDH1^+^ cells (S: 1.2%) compared with their parental counterparts (P: 0.5%, Figure 3A), and the relative ALDH activity was significantly increased (Figure 3B). The expression of stem cell‐related markers, including CD44, Oct‐4A, Sox2 and c‐Myc, was also up‐regulated in CL141 tumour sphere cells (Figure 3C).

Thiostrepton treatment prominently suppressed CL141 tumour spheres formation in a dose‐dependent manner (Figure 3D). This observation was supported by thiostrepton treatment in both parental cells and spheres. Treatment with thiostrepton reduced the expression of CSC markers, including CD133, Oct‐4A and Nanog, as well as suppressed metastasis‐associated Src signalling (Figure 3E).

Furthermore, a stable CSC‐like A549‐ON cells was used to validate the anti‐CSC and anti‐EMT ability of thiostrepton. Thiostrepton treatment significantly decreased expression of Oct‐4A and Nanog and up‐regulated expression of E‐cadherin (Figure 3F), supporting the hypothesis that thiostrepton is a potential anti‐CSC agent.

### 3.4 Thiostrepton and gemcitabine synergistically suppresses NSCLC tumour sphere formation

**Figure 4:**
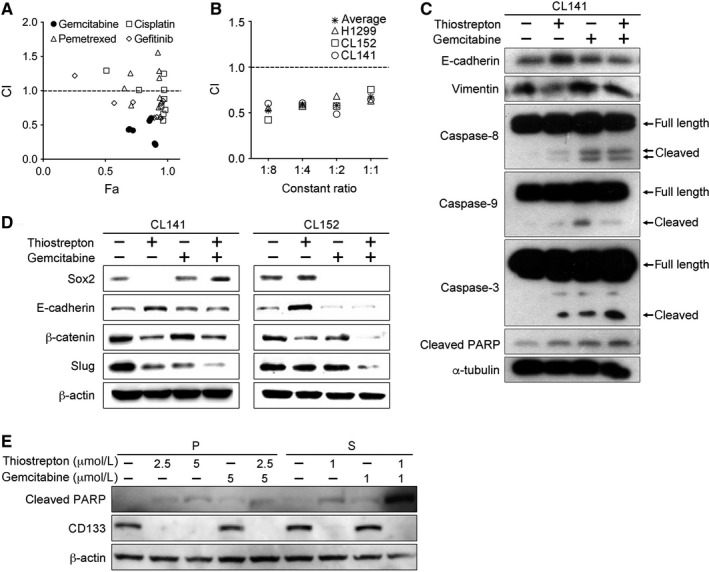
Thiostrepton combined with anti‐cancer drugs exhibits a synergistic effect on NSCLC growth. A, Fa‐CI plot represents the CI values and the Fa (fraction affected, cytotoxic effect) at different concentrations of thiostrepton (1.25, 2.5, and 5 μmol/L) in combination with different chemotherapeutic drugs (pemetrexed, cisplatin, gemcitabine) or gefitinib (5, 10, and 20 μmol/L) for 48 h in CL152 cells. A combination of thiostrepton and gemcitabine showed the highest synergistic effect among the combinations. The data were analysed by CompuSyn software. B, Thiostrepton was combined with gemcitabine at different dosing ratios (1:1, 1:2, 1:4 or 1:8) in CL141, CL152 and H1299 cells. All combinations demonstrated synergistic anti‐NSCLC effects. C, Western blotting assays for CL141 cells treated with thiostrepton and/or gemcitabine. The combination of thiostrepton and gemcitabine effectively reversed EMT and induced apoptosis. D, Comparative Western blotting assays of CL141 and CL152 cells with treatments of thiostrepton, gemcitabine, or thiostrepton plus gemcitabine. Thiostrepton treatment significantly suppressed the expression of β‐catenin (stemness marker) and Slug (EMT marker). When combined with gemcitabine, the suppressive effect was more pronounced in CL152 cells. E, Western blotting assays of CL141 (P) and CL141 spheres (S) treated with thiostrepton and/or gemcitabine. The combination of thiostrepton and gemcitabine effectively suppressed the elevated CD133 expression in the CL141 spheres and induced the apoptotic marker‐cleaved PARP

Thiostrepton in combination with gemcitabine, cisplatin, pemetrexed or gefitinib reduced NSCLC cell viabilities synergistically (Figure 4A). The obtained average CI values from thiostrepton in combination with gemcitabine, gefitinib, cisplatin and pemetrexed were 0.41 ± 0.16, 0.80 ± 0.19, 0.91 ± 0.25 and 1.04 ± 0.30, respectively. This indicates that thiostrepton plus gemcitabine showed the best synergistic effect among these combinations. In addition, the CI values in different ratios (1:1, 1:2, 1:4 and 1:8) all showed synergistic effects in the CL141, CL152 and H1299 cells (Figure 4B).

CD133 and Oct‐4A, the CSC markers, have been suggested as protectors for cancer cells from apoptosis induced by chemotherapeutic agents.^25^, ^26^, ^36^ Significantly increased apoptosis‐related proteins—cleaved caspase‐3 and cleaved PARP—were observed in cells treated with gemcitabine plus thiostrepton (Figure 4C). Apoptosis induced by gemcitabine plus thiostrepton was more significant compared with that induced by gemcitabine or thiostrepton alone (Figure 4C).

Furthermore, the combination of thiostrepton and gemcitabine suppressed expression of stemness markers, such as Sox2, β‐catenin and EMT transcription factor Slug in both CL141 and CL152 cells (Figure 4D), indicating that the combination of thiostrepton and gemcitabine suppresses stemness‐ and EMT‐related profiles in both adenocarcinoma and squamous cell carcinoma cells. This finding suggests thiostrepton could reduce cancer stem‐like cell populations and decrease the risk of distant metastasis.

We also characterized the combination treatment on CL141 parental cells and tumour spheres. Thiostrepton, with or without gemcitabine, was able to suppress CD133 expression, whereas gemcitabine treatment did not (Figure 4E). Importantly, CL141 spheres responded to a lower concentration of thiostrepton (1 µmol/L), as reflected by significantly decreased CD133 levels (Figure 4E). In the presence of thiostrepton, CL141 spheres also responded to gemcitabine at a lower concentration and underwent apoptotic cell death as reflected by the elevated level of cleavage of PARP.

### 3.5 Thiostrepton inhibits NSCLC tumorigenesis and decreases the proportion of CSC in vivo

**Figure 5:**
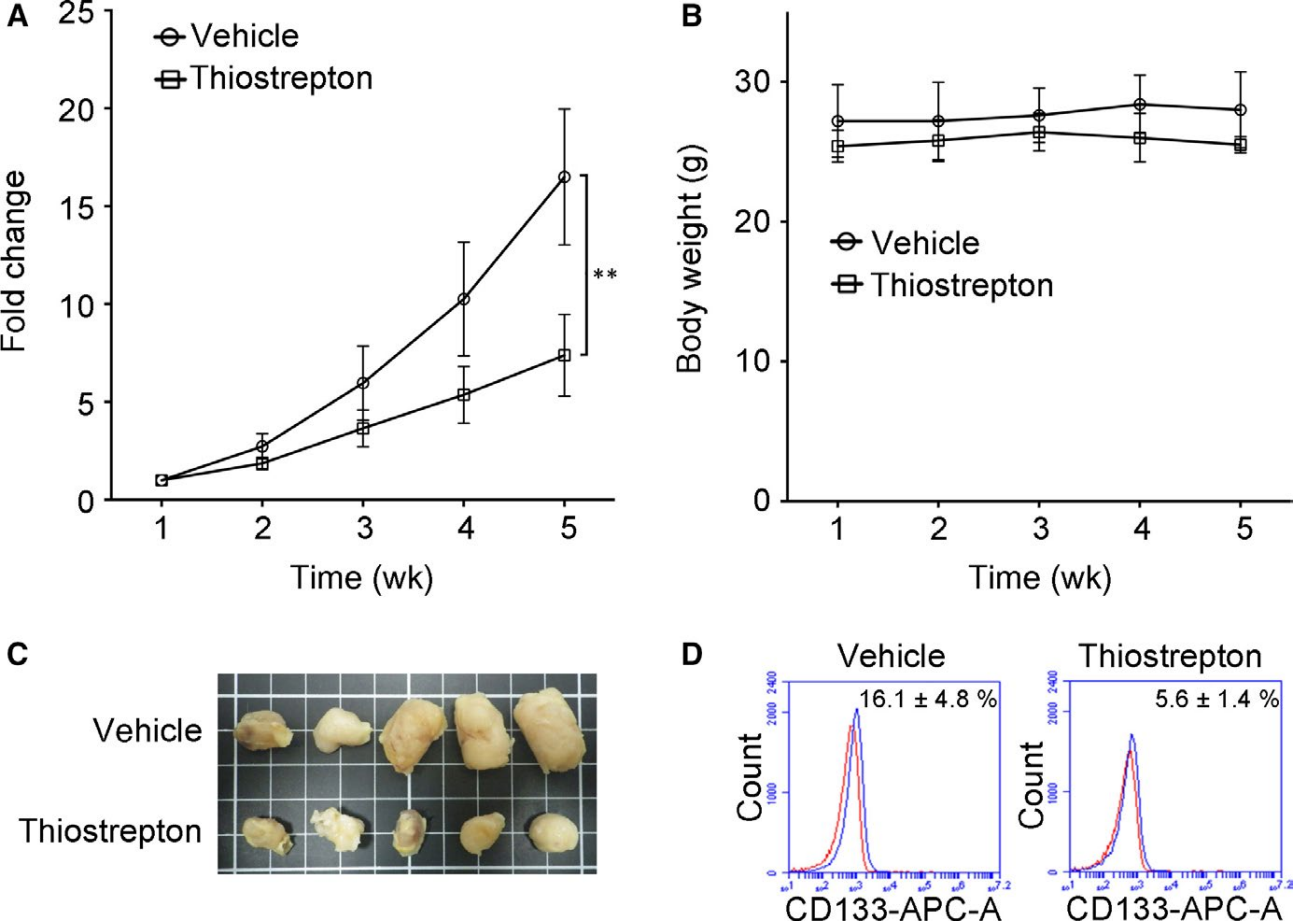
In vivo evaluation of anti‐NSCLC tumour growth effect of thiostrepton. A, H441 tumour‐bearing NOD/SCID mice were used to evaluate the thiostrepton‐mediated suppressive effect on tumour growth. Tumour burden (fold change in the tumour size) is plotted over time. The insert depicts photographs of the tumour samples collected. Thiostrepton treatment (5 mg/kg/d, 5 d/wk, intraperitoneal injection) inhibited H441 tumour growth compared with the vehicle control group after 4 wk of treatment. **P < .01. B, The treatment did not negatively affect the body weight of the animals during the experiment course. C, A photograph of tumours collected from mice in both thiostrepton‐treated and vehicle control group. D, Flow cytometric analysis of tumours demonstrated that thiostrepton treatment significantly decreased the percentage of cells with stem cell marker CD133 expression (presented by the blue peak while the red peak indicates the isotype)

NOD/SCID mice that received daily thiostrepton treatment (n = 5) exhibited a significantly slower tumour growth rate than mice receiving the vehicle treatment (n = 5) at the fifth week, as demonstrated by approximately 2‐fold smaller in tumour size (Figure 5A). The body weight of the mice did not change significantly during the treatment period (Figure 5B). Photographs of harvested tumour samples showed that thiostrepton treatment significantly suppressed tumour growth compared with control counterparts (Figure 5C). Further analysis of the tumour samples revealed that thiostrepton‐treated tumours contained a significantly lower proportion of CD133^+^ cells (5.6%) compared with those of the control group (16.1%) (Figure 5D).

### 3.6 Thiostrepton treatment was associated with an increase in tumour suppressor miR‐98

**Figure 6:**
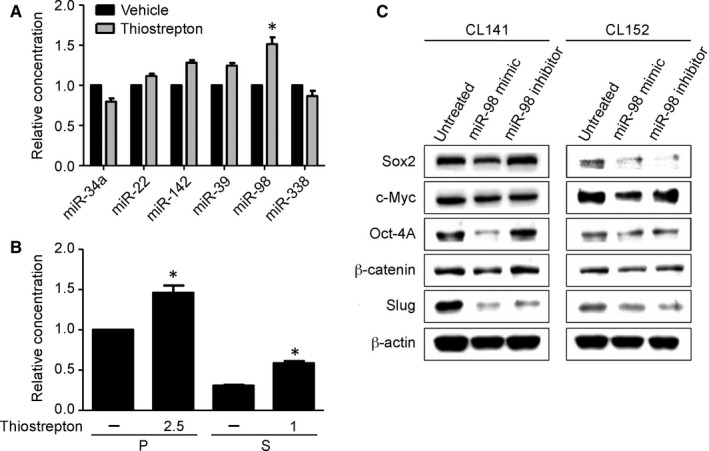
Thiostrepton‐mediated anti‐lung CSC effect is associated with an elevated miR‐98 level. A, A panel of microRNAs was examined in CL141 cells, and the miR‐98 level was the most elevated after thiostrepton treatment. B, Comparative quantitative PCR analysis demonstrated that thiostrepton treatment induced elevation of miR‐98 levels in both CL141 parental (P) and sphere (S) cells. *P < .05 (C) Western blotting assays of miR‐98‐mediated effects in both CL141 and CL152 cells. Cancer stemness/EMT markers, including Sox2, Oct‐4A and β‐catenin, were significantly suppressed when miR‐98 mimic was added. In contrast, when treated with miR‐98 inhibitor, increases in Sox2 and Oct‐4A expression were observed in CL141 cells

MicroRNAs have been extensively characterized and identified in lung cancer and reportedly to play an important promoter or suppressor role, depending on their target genes.^37^, ^38^, ^39^ We examined a panel of microRNAs in response to thiostrepton treatment in CL141 cells, and the miR‐98 level appeared to be elevated by approximately 1.5‐fold (Figure 6A). By comparison, CL141 parental cells contained a significantly higher level of intrinsic miR‐98 than their sphere counterparts (Figure 6B). The level of miR‐98 rose in the wake of thiostrepton treatment in both CL141 parental cells and spheres (Figure 6B).

By increasing miR‐98 (mimic molecules) levels, Sox2, Oct‐4A and β‐catenin were suppressed in both CL141 and CL152 cells (Figure 6C). This was similar to the response to thiostrepton treatment in CL141 cells. Based on the decreased expression of Slug, EMT was considered reversed (Figure 6C). These findings suggest that the suppression of CSC characteristics and EMT by thiostrepton is through up‐regulation of tumour suppressor miR‐98.

## 4 DISCUSSION

In this study, thiostrepton was identified as a potential drug for converting embryonic stem cell‐like gene signatures to those of adult stem cell gene signatures. Through in vitro and in vivo experiments, we demonstrated that thiostrepton effectively inhibited lung CSC growth by the suppressing cancer stemness and expression of EMT genes. A combination of thiostrepton and gemcitabine synergistically suppressed NSCLC cell growth. This exploratory methodology connecting disease, genes and chemical profiles represents an alternative approach to the development of novel therapeutic agents.

In tumour sphere assays, increased EMT potential and expression of CSC markers were showed in NSCLC cells. This finding is in agreement with the previously established hypothesis that enhanced EMT potential is positively associated with the generation of CSCs.^25^, ^40^ In addition, the well‐established CSC marker, CD133, was shown to be associated with up‐regulated expression of EMT‐related genes, including Src and Slug.^41^, ^42^ A recent study reported that FOXM1 directly and constitutively activates Snail in lung adenocarcinoma, thereby promoting cancer metastasis.^43^ Knockdown FOXM1 significantly suppressed EMT progression as well as tumour growth and metastasis.^43^ However, we demonstrated that thiostrepton treatment did not inhibit Snail expression but strongly inhibited Slug expression in NSCLC cells. These findings indicate that the CD133/Src axis, in addition to FOXM1, is a potential therapeutic target for thiostrepton. Thiostrepton treatment not only inhibited CSC markers including CD133, Oct‐4A and Sox2, but also restored E‐cadherin expression, thereby reversing the EMT and generation of cancer spheres. This finding further supports an association between the EMT and CSC.

Ectopic expression of Oct4 and Nanog in lung cancer cells was associated with a significant increase in the percentage of CD133+ cell and mesenchymal cell populations, the ability to form tumour spheres and enhanced drug resistance.^25^ To provide further support the contention that thiostrepton is a potential anti‐CSC agent, we utilized a cell model that A549 cells with overexpression of Oct‐4A/Nanog exhibits enhanced CSC characteristics and EMT potential. Thiostrepton treatment not only significantly suppressed ectopic expression of both Nanog and Oct‐4A in the A549‐ON cells, but also markedly induced epithelial marker E‐cadherin. These findings verify our bioinformatical prediction that thiostrepton treatment is associated with the reversal of CSC gene signatures, EMT status and CSC growth inhibition.

Thiostrepton reportedly enhances sensitivity to platinum in vitro and in vivo.^44^ It has also been reported that cisplatin treatment significantly increases the proportion of CD133^+^ cells through the Notch signal pathway, and CD133^+^ cells are related to an increase in cross‐drug resistance to paclitaxel and doxorubicin.^26^ Therefore, the reduction in CD133+/Oc4‐4A+ cell populations in NSCLC by thiostrepton treatment (Figure 3) provides further support for a role for thiostrepton in combination with chemo‐ and/or target therapeutic agents in preventing drug resistance. Thiostrepton acted synergistically with gemcitabine to inhibit lung cancer cellular viability in this study.

The GSEA results of thiostrepton and thiostrepton plus gemcitabine with concomitant enrichment scores were related to GSE18931 (Table S2), which was evaluated for CSC gene signatures with impacts on clinical outcomes and the pathological features of cancer.^30^ The gene signature of thiostrepton treatment with a positive enrichment score was also significantly related to GSE17215, an anti‐CSC gene signature associated with EMT. Decreased CSCs after drug treatment may prevent tumours from EMT. This finding provides evidence to link the CSC state with EMT. Thiostrepton may follow the same path as salinomycin in reverting M‐type cells back to E‐type cells.^35^ Chemotherapy combined with thiostrepton may therefore be an alternative regimen to improve treatment effectiveness and prolong survival in patients with NSCLC.

Chemotherapy is still a major treatment option for lung squamous cell carcinoma. Thiostrepton in combination with gemcitabine significantly inhibited lung squamous cell carcinoma growth. However, cellular responses towards different treatment regimens varied between adenocarcinoma and squamous cell carcinoma cells. As shown in Figure 4D, thiostrepton alone significantly suppressed Sox2, β‐catenin and Slug expression to a greater extent in CL141 cells than in CL152 cells. While gemcitabine treatment alone appeared to suppress Sox2 expression more significantly in CL152 cells, the suppressive effect of stemness and EMT was more pronounced (Figure 4D). Our previous study showed that thiostrepton can suppress colony and sphere formation and trigger apoptosis of colorectal cancer stem cells in HCT‐15 and HT‐29 cells as well as EMT and chemo‐resistant clones derived from them.^45^ These findings indicate that thiostrepton in combination with chemotherapies may improve treatment effectiveness in lung squamous cell carcinoma.

In this study, an increased level of miR‐98 (a tumour suppressor) was observed in response to thiostrepton treatment. Because miR‐98 is reportedly down‐regulated in many cancer types, its targets, such as Myc, Kras and Wnt signalling, are generally oncogenic.^46^, ^47^, ^48^ The exact targets for thiostrepton‐induced miR‐98 induction in NSCLC warrant further investigation.

In conclusion, thiostrepton was identified as a potential CSC inhibitor for lung cancer using an integrative bioinformatics and pre‐clinical approach. In combination with chemotherapeutic agents, especially gemcitabine, thiostrepton synergistically suppressed NSCLC growth and tumour spheres formation. An elevated miR‐98 level was associated with thiostrepton treatment. These pre‐clinical evidence warrants further clinical studies for thiostrepton in patients with NSCLC.

## CONFLICT OF INTEREST

The authors have no conflict of interest.

## AUTHOR CONTRIBUTIONS

Tse‐Hung Huang, Chi‐Ying F. Huang and Kuan‐Yu Chen designed research; Alexander T. H. Wu, Tai‐Shan Cheng, Kuan‐Ting Lin, Chia‐Jou Lai, Hao‐Wen Hsieh and Peter Mu‐Hsin Chang analysed data; Tse‐Hung Huang, M. Alexander T. H. Wu, Tai‐Shan Cheng, Kuan‐Ting Lin, Chia‐Jou Lai, Hao‐Wen Hsieh and Peter Mu‐Hsin Chang performed research; Tse‐Hung Huang, Alexander T. H. Wu, Tai‐Shan Cheng,Chi‐Ying F. Huang and Kuan‐Yu Chen wrote the paper; Tse‐Hung Huang, Peter Mu‐Hsin Chang and Cheng‐Wen Wu contributed new reagents or analytic tools.

## Data Availability

The data sets generated and analysed during this study are available from the corresponding author on reasonable request.
