# Supplementary material for: In silico identification of thiostrepton as an inhibitor of cancer stem cell growth and an enhancer for chemotherapy in non–small‐cell lung cancer
Source: J Cell Mol Med. 2019 Oct 22;23(12):8184–95. doi: 10.1111/jcmm.14689 (PMC6850923; doi:10.1111/jcmm.14689)
Supplement: Supplementary file 1 [file JCMM-23-8184-s001.docx]

**Supplementary Table 1.** Twenty datasets against cancer stem cell-related gene signatures.

| **Stemness gene signature** | **Reference** |
| --- | --- |
| GSE18931 | [1] |
| GSE17215 | [2] |
| GSE18150 | [3] |
| GSE17375 | [4] |
| CONRAD_GERMLINE_STEM_CELL | [5] |
| GSE16910 | [6] |
| GSE994/GSE1323 | [7-9] |
| RAMALHO_STEMNESS_DN | [10] |
| GSE21336 | [11] |
| GSE6883 | [12] |
| GSE20736 | [13] |
| GSE27313 | [14] |
| GSE28799 | [15] |
| GSE10423 | [16] |
| GSE36821 | [17] |
| GSE25976 | [18] |
| GSE33112 | [19] |
| GSE24460 | [20] |
| GSE24006 | [21] |
| GSE24747 | [22] |

1. **Pece S, Tosoni D, Confalonieri S, Mazzarol G, Vecchi M, Ronzoni S, Bernard L, Viale G, Pelicci PG, Di Fiore PP.** Biological and molecular heterogeneity of breast cancers correlates with their cancer stem cell content. *Cell*. 2010; 140: 62-73.

2. **Gupta PB, Onder TT, Jiang G, Tao K, Kuperwasser C, Weinberg RA, Lander ES.** Identification of selective inhibitors of cancer stem cells by high-throughput screening. *Cell*. 2009; 138: 645-59.

3. **Suva ML, Riggi N, Janiszewska M, Radovanovic I, Provero P, Stehle JC, Baumer K, Le Bitoux MA, Marino D, Cironi L, Marquez VE, Clement V, Stamenkovic I.** EZH2 is essential for glioblastoma cancer stem cell maintenance. *Cancer research*. 2009; 69: 9211-8.

4. **Vermeulen L, De Sousa EMF, van der Heijden M, Cameron K, de Jong JH, Borovski T, Tuynman JB, Todaro M, Merz C, Rodermond H, Sprick MR, Kemper K, Richel DJ, Stassi G, Medema JP.** Wnt activity defines colon cancer stem cells and is regulated by the microenvironment. *Nature cell biology*. 2010; 12: 468-76.

5. **Conrad S, Renninger M, Hennenlotter J, Wiesner T, Just L, Bonin M, Aicher W, Buhring HJ, Mattheus U, Mack A, Wagner HJ, Minger S, Matzkies M, Reppel M, Hescheler J, Sievert KD, Stenzl A, Skutella T.** Generation of pluripotent stem cells from adult human testis. *Nature*. 2008; 456: 344-9.

6. **Tsai ZY, Singh S, Yu SL, Kao LP, Chen BZ, Ho BC, Yang PC, Li SS.** Identification of microRNAs regulated by activin A in human embryonic stem cells. *Journal of cellular biochemistry*. 2010; 109: 93-102.

7. **Spira A, Beane J, Shah V, Liu G, Schembri F, Yang X, Palma J, Brody JS.** Effects of cigarette smoke on the human airway epithelial cell transcriptome. *Proc Natl Acad Sci U S A*. 2004; 101: 10143-8.

8. **Provenzani A, Fronza R, Loreni F, Pascale A, Amadio M, Quattrone A.** Global alterations in mRNA polysomal recruitment in a cell model of colorectal cancer progression to metastasis. *Carcinogenesis*. 2006; 27: 1323-33.

9. **Seo DC, Sung JM, Cho HJ, Yi H, Seo KH, Choi IS, Kim DK, Kim JS, El-Aty AA, Shin HC.** Gene expression profiling of cancer stem cell in human lung adenocarcinoma A549 cells. *Molecular cancer*. 2007; 6: 75.

10. **Ramalho-Santos M, Yoon S, Matsuzaki Y, Mulligan RC, Melton DA.** "Stemness": transcriptional profiling of embryonic and adult stem cells. *Science*. 2002; 298: 597-600.

11. **Ying M, Wang S, Sang Y, Sun P, Lal B, Goodwin CR, Guerrero-Cazares H, Quinones-Hinojosa A, Laterra J, Xia S.** Regulation of glioblastoma stem cells by retinoic acid: role for Notch pathway inhibition. *Oncogene*. 2011; 30: 3454-67.

12. **Liu R, Wang X, Chen GY, Dalerba P, Gurney A, Hoey T, Sherlock G, Lewicki J, Shedden K, Clarke MF.** The prognostic role of a gene signature from tumorigenic breast-cancer cells. *The New England journal of medicine*. 2007; 356: 217-26.

13. **Nogueira L, Ruiz-Ontanon P, Vazquez-Barquero A, Lafarga M, Berciano MT, Aldaz B, Grande L, Casafont I, Segura V, Robles EF, Suarez D, Garcia LF, Martinez-Climent JA, Fernandez-Luna JL.** Blockade of the NFkappaB pathway drives differentiating glioblastoma-initiating cells into senescence both in vitro and in vivo. *Oncogene*. 2011; 30: 3537-48.

14. **Vijayakumar S, Liu G, Rus IA, Yao S, Chen Y, Akiri G, Grumolato L, Aaronson SA.** High-frequency canonical Wnt activation in multiple sarcoma subtypes drives proliferation through a TCF/beta-catenin target gene, CDC25A. *Cancer cell*. 2011; 19: 601-12.

15. **Wang L, Mezencev R, Bowen NJ, Matyunina LV, McDonald JF.** Isolation and characterization of stem-like cells from a human ovarian cancer cell line. *Molecular and cellular biochemistry*. 2012; 363: 257-68.

16. **Wong DJ, Liu H, Ridky TW, Cassarino D, Segal E, Chang HY.** Module map of stem cell genes guides creation of epithelial cancer stem cells. *Cell stem cell*. 2008; 2: 333-44.

17. **Perumal D, Singh S, Yoder SJ, Bloom GC, Chellappan SP.** A novel five gene signature derived from stem-like side population cells predicts overall and recurrence-free survival in NSCLC. *PloS one*. 2012; 7: e43589.

18. **Okuda H, Kobayashi A, Xia B, Watabe M, Pai SK, Hirota S, Xing F, Liu W, Pandey PR, Fukuda K, Modur V, Ghosh A, Wilber A, Watabe K.** Hyaluronan synthase HAS2 promotes tumor progression in bone by stimulating the interaction of breast cancer stem-like cells with macrophages and stromal cells. *Cancer research*. 2012; 72: 537-47.

19. **de Sousa EMF, Colak S, Buikhuisen J, Koster J, Cameron K, de Jong JH, Tuynman JB, Prasetyanti PR, Fessler E, van den Bergh SP, Rodermond H, Dekker E, van der Loos CM, Pals ST, van de Vijver MJ, Versteeg R, Richel DJ, Vermeulen L, Medema JP.** Methylation of cancer-stem-cell-associated Wnt target genes predicts poor prognosis in colorectal cancer patients. *Cell stem cell*. 2011; 9: 476-85.

20. **Calcagno AM, Salcido CD, Gillet JP, Wu CP, Fostel JM, Mumau MD, Gottesman MM, Varticovski L, Ambudkar SV.** Prolonged drug selection of breast cancer cells and enrichment of cancer stem cell characteristics. *J Natl Cancer Inst*. 2010; 102: 1637-52.

21. **Gentles AJ, Plevritis SK, Majeti R, Alizadeh AA.** Association of a leukemic stem cell gene expression signature with clinical outcomes in acute myeloid leukemia. *Jama*. 2010; 304: 2706-15.

22. GEO, <https://www.ncbi.nlm.nih.gov/geo/query/acc.cgi?acc=GSE24747> (Oct 18, 2010)

**Supplementary Table 2.** L1000 gene profiles of thiostrepton and thiostrepton plus gemcitabine compared with anti-CSC or CSC-like signatures from GEO identified by gene set enrichment analysis.

| Based on (compound) | Signature Name | Characters of gene signature | ES | NES | Nominal p-value | FDR |
| --- | --- | --- | --- | --- | --- | --- |
| Thiostrepton | GSE18931 | CSC | -0.6936 | -2.0146 | <0.0001 | <0.0001 |
| Thiostrepton | GSE17215 | Anti-CSC, Anti-EMT | 0.5852 | 1.4360 | 0.0049 | 0.2350 |
| Thiostrepton | GSE18150 | Anti-CSC | 0.7975 | 1.9139 | <0.0001 | <0.0001 |
| Thiostrepton_G | GSE18931 | CSC | -0.5521 | -1.4189 | 0.0216 | 0.0984 |
| Thiostrepton_G | GSE18150 | Anti-CSC | 0.8250 | 1.7505 | 0.0017 | 0.0031 |

ES: Enrichment scores; NES: Normalized enrichment scores; FDR: False discovery rate; Thiostrepton_G: Thiostrepton plus gemcitabine; CSC: cancer stem cell

**Supplementary Table 3.** Cell viability analysis in NSCLC cells treated by thiostrepton.

|  |  |  |  |  |
| --- | --- | --- | --- | --- |
| Cancer type | Cell line | *EGFR* mutation status | SRB assay (IC_50_) (μM) | Clonogenic assay (IC_50_) (μM) |
|  |  |  |  |  |
| Adenocarcinoma | A549 | WT | 3.0 ± 1.0 | ~0.05 |
| Adenocarcinoma | A549-ON | WT | 0.8 ± 0.3 |  |
| Primary Adenocarcinoma | CL141 | WT | 2.7 ± 1.2 | ~0.05 |
| Primary Squamous cell carcinoma | CL152 | WT | 1.6 ± 0.6 | ~0.05 |
| Non-small cell lung cancer | H1299 | WT | 3.7 ± 1.2 | ~0.05 |
| Large cell carcinoma | H460 | WT | 6.9 |  |
| Large cell carcinoma | H460 CD133+ | WT | 1.7 |  |

IC50: half maximal inhibitory concentrations.

A549-ON cells: A549 cells with ectopic co-overexpressed Oct and Nanog.

H460 CD133+ cells: cells infected by CD133 P1 promoter–driven GFP reporter lentivirus and cultured in complete medium.

The GFP+ cells, H460 CD133+ cells, were sorted using a FACSAria cell sorter (BD Biosciences).

WT: wild type.
